# Detection of Major Depressive Disorder from Functional Magnetic Resonance Imaging Using Regional Homogeneity and Feature/Sample Selective Evolving Voting Ensemble Approaches

**DOI:** 10.3390/jimaging11070238

**Published:** 2025-07-14

**Authors:** Bindiya A. R., B. S. Mahanand, Vasily Sachnev, DIRECT Consortium

**Affiliations:** 1 Department of Computer Science and Engineering, Sri Jayachamarajendra College of Engineering, JSS Science and Technology University, Mysuru 570006, Karnataka, India; bindiyaramesh@sjce.ac.in; 2 Department of Information Science and Engineering, Sri Jayachamarajendra College of Engineering, JSS Science and Technology University, Mysuru 570006, Karnataka, India; 3 Department of Information, Communications and Electronic Engineering, Catholic University of Korea, Bucheon 14662, Republic of Korea

**Keywords:** major depressive disorder, functional magnetic resonance imaging, regional homogeneity, evolving voting ensemble

## Abstract

Major depressive disorder is a mental illness characterized by persistent sadness or loss of interest that affects a person’s daily life. Early detection of this disorder is crucial for providing timely and effective treatment. Neuroimaging modalities, namely, functional magnetic resonance imaging, can be used to identify changes in brain regions related to major depressive disorder. In this study, regional homogeneity images, one of the derivative of functional magnetic resonance imaging is employed to detect major depressive disorder using the proposed feature/sample evolving voting ensemble approach. A total of 2380 subjects consisting of 1104 healthy controls and 1276 patients with major depressive disorder from Rest-meta-MDD consortium are studied. Regional homogeneity features from 90 regions are extracted using automated anatomical labeling template. These regional homogeneity features are then fed as an input to the proposed feature/sample selective evolving voting ensemble for classification. The proposed approach achieves an accuracy of 91.93%, and discriminative features obtained from the classifier are used to identify brain regions which may be responsible for major depressive disorder. A total of nine brain regions, namely, left superior temporal gyrus, left postcentral gyrus, left anterior cingulate gyrus, right inferior parietal lobule, right superior medial frontal gyrus, left lingual gyrus, right putamen, left fusiform gyrus, and left middle temporal gyrus, are identified. This study clearly indicates that these brain regions play a critical role in detecting major depressive disorder.

## 1. Introduction

Mental health refers to an individual’s emotional, psychological, and social well-being, and one of the most common mental health disorders in the world is major depressive disorder (MDD). As per the recent world health organization report on mental health, approximately 280 million people globally suffer from MDD, and the report emphasizes the importance of integrating mental health services into primary healthcare [1]. Symptoms of MDD include persistent sadness, loss of energy, restlessness, feelings of worthlessness, guilt, thoughts of self-harm, and loss of interest in activities. Several factors like chemical imbalance, life events, and genetics may contribute to major depressive disorder. MDD can be diagnosed by a psychiatrist or psychologist through clinical interviews, medical history, and standardized screening tools such as the diagnostic statistical manual and international classification of diseases [2].

At present, neuroimaging modalities, namely, functional magnetic resonance imaging (fMRI), are being widely employed in the detection of major depressive disorder [3]. fMRI is a non-invasive technique that detects brain activity by identifying changes in blood oxygen levels. When a specific area of the brain becomes active, it requires more oxygen and blood flow to that region increases. The difference in oxygen levels between oxygenated and deoxygenated blood affects the properties of the blood, which can be detected using an fMRI scanner [4,5]. For better understanding of brain changes, fMRI indices such as regional homogeneity (ReHo), amplitude low-frequency fluctuation (ALFF) and fractional ALFF (fALFF), degree centrality (DC), and voxel-mirrored homo-topic connectivity (VMHC) are normally derived [6]. Among these indices, the regional homogeneity approach measures the similarity of the given voxel with the neighboring voxels using Kendall’s coefficient of concordance to detect changes in brain [7].

Advances in machine learning approaches have allowed researchers to employ functional magnetic resonance imaging to detect major depressive disorder. These approaches process high-dimensional neuroimaging data to extract relevant features, which are then used to classify individuals as having MDD or being a healthy control. A recent study by Peishan et al. employed a transformer encoder model, which is a deep learning model for the classification of MDD using functional connectivity features, and obtained an accuracy of 68.61%. In addition, the study also revealed brain regions namely frontal gyri and cerebral cortex which are relevant for MDD [8]. Another study conducted by Selene Gallo et al. demonstrates the functional connectivity features to classify MDD with healthy controls (HCs) by using support vector machine (SVM) with an accuracy of 62% [9]. A study conducted by Yamashita using functional connectivity features classified MDD by employing logistic regression method to build a generalizable brain network and obtained an accuracy of 70%. Additionally, the study identified brain changes in the default mode and attention networks that are associated with MDD [10]. Yoshida et al. used partial least squares path modeling and detected MDD from 123 subjects and obtained an accuracy of 80%. The study identified right superior frontal gyrus and superior motor area regions which are associated with depression [11]. Mwangi et al. in their study, combined functional, morphological, and anatomical features to classify MDD using random forest and obtained 90.3% accuracy. Additionally, the features predicted suicide risks of patients with MDD [12]. In a study by Liu and colleagues, researchers used machine learning approaches to analyze brain scans from both large, multi-site datasets and smaller, single-site datasets. They revealed that the multi-site dataset achieved a balanced accuracy of 62%, while the smaller single-site datasets reported accuracies over 90% [13].

From the literature, it is observed that traditional machine learning approaches perform better for smaller datasets, and there exists challenges to achieving better results from a large multi-site dataset. Hence, this study proposes a feature/sample evolving voting ensemble method to classify major depressive disorder from the REST-meta-MDD dataset. The fsEVE involves a new feature/sample selection genetic algorithm and modified evolving voting ensemble methods to classify MDD and HCs. Further, the brain regions which may be responsible for MDD are identified.

The organization of this paper is as follows: Section 2 presents dataset and the proposed feature/sample selective voting ensemble classifier of the study. Section 3 presents the results and discussions of the study, and Section 4 summarizes the major conclusions of the study.

## 2. Materials and Methods

### 2.1. Dataset and Preprocessing

The dataset used in this study is obtained from the REST-meta-MDD direct consortium, which is collected from 17 different sites and 25 hospitals [14]. A total of 2380 subjects consisting of 1276 major depressive disorder patients and 1104 healthy controls are used for this study. Among the MDD subjects, 813 are female and 463 are male, whereas within the HCs, 642 are female and 462 are male. Detailed demographic information of the dataset is presented in Table 1. As the raw image data is subjected to head motion and slice timing variations, the fMRI images are preprocessed using data processing assistant for resting-state fMRI (DPARSF) tool [15]. All resting-state fMRI scans in the consortium are processed at each site using the same DPARSF protocol described in [14]. In this study, already preprocessed data available in REST-meta-MDD consortium is used. The preprocessing pipeline employed by the Rest-meta-MDD project is as follows. The preprocessing was initiated by removing the first 10 volumes of each scan to allow the MRI signal to stabilize, followed by slice-timing correction, applied to adjust the differences in the timing of slice acquisition. Next, a rigid body transformation was applied to correct head motion by aligning each subject’s image time series. Each participant’s T1-weighted anatomical image was then aligned to the mean functional image using a six-degree-of-freedom linear transformation without resampling. Then, the image was segmented into gray matter, white matter, and cerebrospinal fluid. The images were normalized to monuments of national importance (MNI) standard space using the diffeomorphic anatomical registration through exponentiated lie algebra (DARTEL) algorithm, which involved calculating and applying the necessary transformations from native space. After normalization, nuisance signals—including the friston 24-parameter head motion model—as well as white matter and cerebrospinal fluid signals were removed from the data using linear regression. A linear trend regressor was also included to account for slow drifts in the BOLD signal. Finally, temporal band-pass filtering between 0.01 and 0.1 Hz was applied to all time series to retain the frequency range most relevant for resting-state analyses. Regional homogeneity (ReHo) images are the data derived from functional magnetic resonance imaging. ReHo features from 90 regions are extracted using automated anatomical labeling (AAL) template version 1 [16]. The AAL template consists of 90 anatomically defined regions from cerebrum (45 per hemisphere). These 90 brain regions were further not divided or merged. A total of 47,636 ReHo features from each regional homogeneity image is obtained with dimension of 2380 × 47,636. These ReHo features are then used as an input to the proposed feature/sample evolving voting ensemble approach.

### 2.2. The Feature/Sample Selective Evolving Voting Ensembler

As the dimension of the features (2380 × 47,636) is very large in this study, traditional machine learning approaches may not efficiently classify MDD. Ensemble approaches are recently used to handle high dimensional data, and there are several approaches, such as stacking, boosting, and bagging [17,18]. The proposed “Feature/Sample Selective Evolving Voting Ensemble” approach is an example of ensemble learning which aims to accurately detect major depressive disorder.

The fsEVE typically uses majority voting, where predictions from base models are aggregated to make a final decision. Unlike static ensembles, fsEVE evolves over time, adapting by adding and removing models based on their performance. This adaptive process ensures that the ensemble is consistently based on the most effective models. Extreme learning machine (ELM) is selected as the base model for fsEVE. ELM is a single hidden layer feed-forward neural network, where input weights and bias are assigned randomly and output weights are computed analytically [19]. In this study, ELM is preferred over other traditional models to efficiently handle high-dimensional data (2380 × 47,636), enabling rapid training and accurate detection of major depressive disorder.

**Figure 1:**
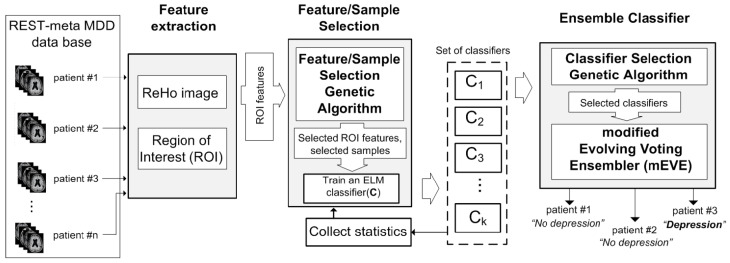
Feature /sample selective evolving voting ensembler framework.

The framework of the proposed feature/sample selective evolving voting ensemble (fsEVE) is illustrated in Figure 1. The fsEVE framework consists of three main components, namely, “Feature Extraction,” “Feature Selection,” and the final “Ensemble Classifier”.

The “Feature selection” component selects the most relevant features for MDD classification and creates a set of classifiers for the next ensemble. The component utilizes the “Feature/Sample Selection Genetic Algorithm” to select sets of ReHo features and samples for training to build a set of ELM classifiers. The “Feature/Sample selection Genetic Algorithm” selects samples based on the unique measures called “Sample Hardness” and “Classifier cost”. “Sample Hardness” is calculated by using statistics collected from previously built ELM classifiers and indicates how easy or hard each sample can be classified. Finally, “hard” samples have a higher chance of being selected by a “Feature/Sample selection Genetic Algorithm” to contribute to the next genetic algorithm generations. ELM classifiers trained by using hard samples significantly improve the overall accuracy of the modified evolving vote ensembler with “Classifier cost” as fitness function. Finally, the “Feature/Sample Selection Genetic Algorithm” builds 2000 ELM classifiers.

The “Ensemble classifier” component unifies the “Classifier Selection Genetic Algorithm (CSGA)” and the modified evolving voting ensemble (mEVE), which is used to build a final ensemble classifier for accurately detecting major depressive disorder. The “Classifier Selection Genetic Algorithm” searches for the initial set of classifiers (among 2000 classifiers generated by “Feature/Sample Selection Genetic Algorithm”) for further improvement using mEVE. By adding or subtracting ELM classifiers iteratively, mEVE adjusts the set of classifiers until the overall training accuracy significantly increases. mEVE uses the majority vote strategy in order to decide on each sample.

#### 2.2.1. The Feature/Sample Selection Genetic Algorithm

The “Feature/Sample Selection Genetic Algorithm” is a modified version of the classic Genetic Algorithm (GA) designed specifically to find the ReHo features and samples for training and to build a set of ELM classifiers for a further ensemble. The proposed “Feature/Sample Selection Genetic Algorithm” uses the unique concept of adaptive sample hardness measure and Classifier Cost, as a fitness. In the processing step, a set of 100 ELM classifiers with randomly picked samples is created. The “sample hardness” measure is calculated based on the number of ELM classifiers (among 100), which correctly classify the current sample. The sample is considered as “Hard” if the number of ELM classifiers that correctly classify this sample is minimum or equal to 0. The sample is considered as “Simple” if this sample was classified by the maximum number of ELM classifiers.

The “Classifier cost” is another measure that accumulates weighted “Sample hardness” and highlights the importance of each trained ELM classifier in the ensemble model. The ELM classifier with a high “Classifier cost” has the maximum number of “Hard” samples that are classified correctly. Training and testing accuracies of such ELM classifier may not be high, because less number of samples are classified correctly. However, such an ELM classifier may contribute more to the ensemble classifier. In the proposed Feature/Sample Selective Evolving Voting Ensembler, the “Classifier cost” is used as the fitness function for the Feature Selection Genetic Algorithm. The statistic collection procedure based on “Sample hardness” and “Classifier cost” is presented as follows:

(1) Train set of 100 ELM classifiers using randomly selected features.
(2) Build a decision matrix $M_{100\times 47,636}$, where each matrix element $M_{i,j}$ is either 0 or 1, 100 is the number of initial ELM classifiers, and 47,636 is the number of features. $M_{i,j}=0$ refers to misclassified samples, and $M_{i,j}=1$ refers to the correct classification of the j-th sample for the i-th classifier.
(3) Compute opinion score for each sample $Op_{j}=\sum_{i}M_{i,j}$. Opinion score defines the number of ELM classifiers that correctly classify the j-th sample.
(4) “Sample hardness” is computed as follows:
  (1) $$H_{j}=D\cdot e^{-\frac{r_{j}^{x}}{n}}$$
  where $r_{j}$ is the rank of the j-th sample in the sorted row of the opinion scores Op sorted in ascending order. $H_{r}$ is the sample hardness of the sample with rank *r*, *D* is a scaling factor, n=47,636 is the number of samples, and *x* is the exponential constant. In this simulation, constants x=1.2 and D=200 are selected in the way to maximize the effect of “sample hardness” for further ensemble. The “sample hardness” is higher for samples with lower opinion scores and vice versa.
(5) “Classifier Cost” $C_{p}$ for p-th classifier is calculated based on the “Sample hardness” as follows:
  (2) $$C_{p}=\sum_{q=1}^{n}H_{q}$$
  where
  (3) $$\begin{array}{c} H_{q}=\left\{\begin{array}{cc} 0 & \mathrm{if}\;M(p,q)=0,\\ H_{q} & \mathrm{if}\;M(p,q)=1 \end{array}\right. \end{array}$$

The “Feature/Sample selection genetic algorithm” selects “Hard” samples for training with high “sample probability”, another measure calculated based on the above-named “sample hardness”. “Sample probability” defines the probability of each sample to contribute to the training process so that “Hard” samples have a high chance of being selected, and “Simple” samples have a minimum (not zero) chance of being selected. The “sample probability” for *j*-th sample is computed as follows:

(4) $$p_{j}=\frac{H_{j}}{max(H)}$$

The training set Tra is built as follows:

(5) $$\begin{array}{c} {Tra}_{i}=\left\{\begin{array}{cc} & sample_{j}\;\mathrm{is}\;\mathrm{picked},\;\mathrm{if}\;p_{j}<r\\ & sample_{j}\;\mathrm{is}\;\mathrm{skipped},\;\mathrm{otherwise} \end{array}\right. \end{array}$$

where *i* defines the *i*-th sample in the training set, *j* defines the *j*-th sample among all available samples for training, and r∈[0,1] is the random number.

The proposed “Feature/Sample Selection Genetic Algorithm” and “Classifier Selection Genetic Algorithm” are variations of the traditional genetic algorithm (GA), which is an effective iterative heuristic optimization approach widely used in various areas of engineering and science [20].

The ”Feature/Sample Selection Genetic Algorithm” contains six main steps: “Initialization”, “Genes representation”, “Genetic operators, which are Crossover and Mutation”, “Selection procedure”, “Fitness function”, and “Termination criteria”.

Initialization: In this step, the Sample Hardness, Classifier Cost, and Sample Probability are computed in accordance with Equations (1), (2), and (4).

Genes representation vector *G*: In the proposed “Feature/Sample Selection Genetic Algorithm,” the training data is built from the set of 47,636 ReHo features extracted from the ReHo images. Each ReHo feature is presented as a gene and is coded as a binary coefficient “1” or “0” in the genes representation vector *G*, where binary “1” indicates the selected ReHo feature, and otherwise, the ReHo feature is skipped. The genes representation vector *G*, together with the method described in Equation (5), forms the training set used to train the ELM classifier.

Selection procedure: It is required to provide each gene (ReHo feature) with a unique opportunity to contribute to the following generation within the GA framework. Higher fitness genes (ReHo features) are more likely to develop a new strategy, and vice versa. For selection, the normalized geometric ranking algorithm is employed [21].

Genetic operators: Crossover and mutation are the main genetic operators in the genetic algorithm framework. Both operators copy the gene adaptation process from nature, where future generations of life have a higher chance of surviving in extreme environments. In the genetic algorithm, the crossover and mutation are chosen and/or redesigned based on the complexity of the problem.

Crossover is a genetic operator that recombines and transfers genetic information between generations of life forms. Crossover randomly enables or disables features coded by genes for survival. In the proposed “Feature/Sample Selection Genetic Algorithm”, the novel balanced single-point crossover specifically designed for the detection of major depressive disorder. Compared to classical arithmetic, heuristic, or binary crossover, the single-point crossover guarantees diversity of the gene recombination process and avoids the local optimum dilemma. The single-point crossover deals with gene representation vectors *G* is responsible for selecting ReHo features and training the ELM classifiers.

The framework of the proposed balanced single-point crossover is presented in Algorithm 1. For two given gene representation vectors $G_{1}$ and $G_{2}$, define two position vectors of all binary “1” $Pos_{1}$ and $Pos_{2}$ using function “Pos”. Define position vector $P_{same}$ or common positions of binary“1” (referred to the selected ReHo features common for $G_{1}$ and $G_{2}$) and Pd or different positions of binary ”1“ using functions “Getsame” and “Getdiff”. Divide $Pd=Pd_{1}\cup Pd_{2}$ into two disjoint vectors $Pd_{1}$ and $Pd_{2}$ randomly using function “split(Pd,α)”, where α∈[0.3,0.7] is a split coefficient. Build two new position vectors $P_{1}^{new}$ and $P_{2}^{new}$ and corresponding gene representations vectors $G_{1}^{new}$ and $G_{2}^{new}$ using inverse function “${\mathrm{Pos}}^{-1}$”, which converts position into the binary vector. Train two ELM classifiers using ReHo features highlighted by $G_{1}^{new}$ and $G_{2}^{new}$ and samples selected by the method presented in Equation (5). The function “fitness” returns the training efficiency of the ELM classifier. The gene representations vector with higher fitness $G^{new}$ returns as a result of the “Balanced single-point crossover” algorithm.
**Algorithm 1** Balanced Single-Point Crossover$G^{new}=BSPcross\left(G_{1},G_{2}\right)$:$P_{1}=Pos\left(G_{1}\right)$$P_{2}=Pos\left(G_{2}\right)$$P_{same}=Getsame\left(P_{1},P_{2}\right)$$P_{diff}=Getdiff\left(P_{1},P_{2}\right)$$Pd_{1},Pd_{2}=split\left(Pd,\alpha \right)$$P_{1}^{new}=P_{same}\cup Pd_{1},G_{1}^{new}=Pos^{-1}\left(P_{1}^{new}\right)$$P_{2}^{new}=P_{same}\cup Pd_{2},G_{2}^{new}=Pos^{-1}\left(P_{2}^{new}\right)$$fit_{1}=fitness\left(G_{1}^{new}\right)$$fit_{2}=fitness\left(G_{2}^{new}\right)$**if **$fit_{1}>fit_{2}$** then**    $G^{new}=G_{1}^{new}$**else if **$fit_{1}<=fit_{2}$** then**    $G^{new}=G_{2}^{new}$**end if**

Mutation is another important genetic operator in the genetic algorithm. Mutation randomly replaces or reconfigures genes in a way to disrupt the crossover operator. Mutation may deploy new properties to the solutions field, which may be difficult to find using crossover operator. The combination of mutation and crossover guarantees the convergence of the genetic algorithm and resolves the local minima problems. In the “Feature/Sample Selection Genetic Algorithm” and “Classifier Selection Genetic Algorithm”, the mutation randomly picks and replaces one gene representation vector with another randomly generated gene representation vector $G_{mut}$ with the same number of binary “1”.

Fitness Function is a method to numerically estimate each gene representation vector *G* generated by crossover or mutation. In the proposed “Feature/Sample Selection Genetic Algorithm”, the classifier cost (refer Equation (2)) is used as a fitness function. Gene representation vectors with higher fitness contain more relevant features that may be responsible for MDD. Combination of ELM classifiers created by using a set of gene representation vectors *G* with higher fitness builds a more efficient pool of ELM classifiers for further ensemble.

Termination Criterion stops a genetic algorithm when the method cannot improve a fitness during 10% from a total number of generations.

Both “Feature/Sample Selection Genetic Algorithm” and “Classifier Selection Genetic Algorithm” use the proposed balanced single-point crossover, mutation, and the normalized geometric ranking method for selection [21].

#### 2.2.2. Modified Evolving Voting Ensembler

The proposed “Modified Evolving Voting Ensemble” is an iterative growing/deleting approach to search for a pool of classifiers for further ensemble. mEVE processes a large initial set of accessible classifiers and searches for an optimal pool of classifiers for the voting ensemble with maximum accuracy. In this study, the evolving voting ensemble approach previously used for steganalysis is modified to detect major depressive disorder [22].

The mEVE starts from an odd set of ELM classifiers selected by “Classifier Selection Genetic Algorithm” from a set of 2000 ELM classifiers *S* created by the “Feature/Sample Selection Genetic Algorithm”. The modified evolving voting ensemble steps are presented in Algorithm 2.
**Algorithm 2** Modified Evolving Voting EnsemblerGiven a large pool *S* of *N* available ELM classifiers for ensembling and a pool $s_{0}$ of *n* (odd) ELM classifiers selected from *S*.(1) Compute accuracy $\eta_{0}$ for $s_{0}$ using Equation (6).(2) “Remove-remove” strategy:(2.a) Remove ELM classifier from $s_{0}$ one by one, build *n* sets of classifiers $s_{R}^{k}$ of size n−1, where the *k* is the position of the removed classifier.(2.b) For each set of classifiers $s_{R}^{k}$ compute the number of neutral samples $n_{e}$ using Equation (8). Pick 5 sets $s_{R}^{k(1)}$,$s_{R}^{k(2)}$,...,$s_{R}^{k(5)}$ with the maximum number of neutral samples $n_{e}$.(2.c) Remove another classifier from $s_{R}^{k(i)}$, build 5·(n−1) sets of classifiers $s_{RR}^{k(i),m}$ with n−2 classifiers, where the *m* is the position of the second removed classifier.(2.d) For each $s_{RR}^{k(i),m}$ compute accuracy η using Equation (6), pick a set $s_{RR}$ with the maximum accuracy $\eta_{RR}$.(3) “Remove-Add” strategy:(3.a) and (3.b) Repeat steps 2.a, 2.b(3.c) Add one by one a classifier from set *S* to $s_{R}^{k(1)}$,$s_{R}^{k(2)}$,...,$s_{R}^{k(5)}$, build 5·N set of classifier $s_{RA}^{k\left(i\right),m_{A}}$ with *n* classifiers, where the $m_{A}$ is the position of the inserted classifier.(3.d) For each $s_{RA}^{k\left(i\right),m_{A}}$ compute accuracy η using Equation (6), pick a set $s_{RA}$ with the maximum accuracy $\eta_{RA}$.(4) “Add-Add” strategy:(4.a) Add ELM classifier from *S* one by one, build *n* sets of classifiers $s_{A}^{z}$ of size n+1, where the *z* is the position of the inserted classifier.(4.b) For each set of classifiers $s_{A}^{z}$ compute the number of neutral samples $n_{e}$ using Equation (8). Pick 5 sets $s_{A}^{z(1)}$,$s_{A}^{z(2)}$,...,$s_{A}^{z(5)}$ with the maximum number of neutral samples $n_{e}$.(4.c) Add another classifier to $s_{A}^{z(i)}$, build 5·N sets of classifiers $s_{AA}^{z\left(i\right),y_{A}}$ with *n* classifiers, where the *y* is the position of the second inserted classifier.(4.d) For each $s_{AA}^{z(i),y}$ compute accuracy η using Equation (6), pick a set $s_{AA}$ with the maximum accuracy $\eta_{AA}$.(5) “Add-Remove” strategy:(5.a) and (5.b) Repeat steps 4.a, 4.b(5.c) Remove classifier from $s_{A}^{z(1)}$,$s_{A}^{z(2)}$,...,$s_{A}^{z(5)}$, build 5·n set of classifier $s_{AR}^{z\left(i\right),y_{R}}$ with *n* classifiers, where the $y_{R}$ is the position of the removed classifier.(5.d) For each $s_{AR}^{z\left(i\right),y_{R}}$ compute accuracy η using Equation (6), pick a set $s_{AR}$ with the maximum accuracy $\eta_{AR}$.(6) Find the most successful strategy among “Remove-Remove”, “Remove-Add”, “Add-Add”, and “Add-Remove” with the maximum accuracy $\eta_{m}=max\left(\eta_{RR},\eta_{RA},\eta_{AA},\eta_{AR}\right)$. Replace $s_{0}$ to $s_{RR}$,$s_{RA}$,$s_{AA}$,$s_{AR}$ according to $\eta_{m}$.**if **$\eta_{m}>\eta_{0}$** then**    Repeat steps 2,3,4,5,6.**else if **$\eta_{m}=\eta_{0}$** then**    Stop and return $s_{0}$.**end if**

The accuracy η of the modified evolving voting ensembler is calculated as follows:

(6) $$\eta =\frac{1}{n}\sum_{q=1}^{n}J_{q}$$

where

(7) $$\begin{array}{c} J_{q}=\left\{\begin{array}{cc} & 0,\;\mathrm{if}\;\sum_{p=1}^{n}M\left(p,q\right)>\frac{n}{2}\\ & 1,\;\mathrm{if}\;\sum_{p=1}^{n}M\left(p,q\right)<\frac{n}{2} \end{array}\right. \end{array}$$

The number of neutral samples $n_{e}$ for any set of classifiers $s_{0}$ with *n* classifiers is computed as follows:

(8) $$n_{e}=\sum_{q=1}^{n}G_{q}$$

where

(9) $$\begin{array}{c} G_{q}=\left\{\begin{array}{cc} & 1,\;\mathrm{if}\;\sum_{p=1}^{n}M\left(p,q\right)=\frac{n}{2}\\ & 0,\;\mathrm{otherwise} \end{array}\right. \end{array}$$

Starting with any initial pool $s_{0}$ of ELM classifiers, the mEVE iteratively adds or eliminates classifiers before stopping when the optimal set of ELM classifiers has been chosen. The properties of the large pool of all possible ELM classifiers *S* and the beginning pool $s_{0}$ chosen by the “Classifier Selection Genetic Algorithm” determine the performance of the proposed mEVE. If more “hard” samples are accurately classified by the available classifiers, mEVE performs better. In order to classify “hard” samples more accurately using mEVE, the proposed “Feature/Sample Selection Genetic Algorithm” provides enough ELM classifiers.

## 3. Results and Discussion

### 3.1. Experimental Results

The experiments are conducted using 2380 subjects, and they are split into training (75%) and testing (25%) sets 5 times randomly. For each random split, a full set of experiments has been conducted, i.e., a set of 2000 ELM classifiers has been created by using the “Feature/Sample Selection Genetic Algorithm”, then a ”Classifier Selection Genetic Algorithm“ has been used to build many initial sets of the ELM classifiers, which were iteratively improved by mEVE. The ”Feature/Sample Selection Genetic Algorithm” has a population size of 200, a crossover probability of 70%, mutation probability of 30% with the fitness function as a “Classifier cost”. The “Classifier Selection Genetic Algorithm” has the population size of 100, a crossover probability of 75%, mutation probability of 25% with the fitness function as the training accuracy of the mEVE.

Table 2 presents the performance evaluation of the proposed fsEVE approach and information on the final ensemble models with maximum performance for each split. The best ensemble model (“split 5”) contains 467 ELM classifiers with a maximum/minimum training accuracy of 60.34%/50.93%. ELM classifiers have low training and testing accuracies due to the effect of “Classifier Cost”, which was used as a fitness for “Feature/Sample Selection Genetic Algorithm”. However, a created set of ELM classifiers has unique properties to classify more “hard” samples and to construct a very accurate ensemble classifier. The “Classifier Selection Genetic Algorithm” found the best ensemble model after 673 generations. Training accuracy for the final ensemble model is 99.61%, while testing accuracy is 91.93%. The training F1 score for the final ensemble is 0.99 and the testing F1 score is 0.98. The results of the experiment clearly indicate that the random splits do not significantly affect the performance of the proposed feature/sample selective evolving voting ensembler.

**Figure 2:**
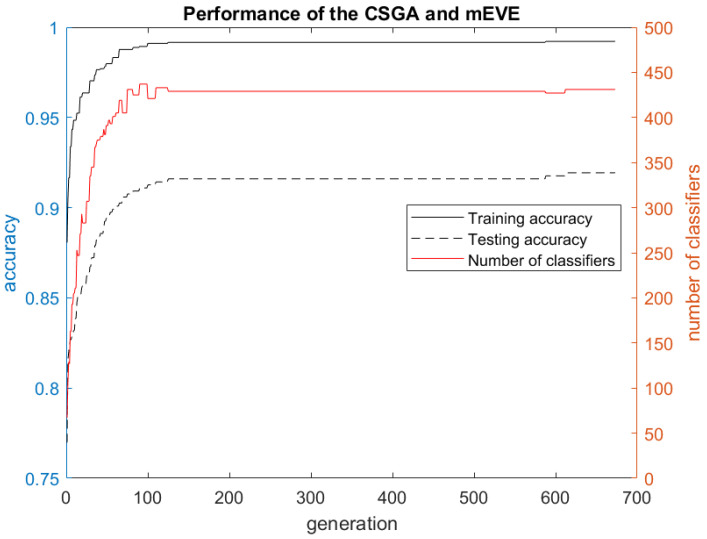
The performance evaluation of the “Classifier Selection Genetic Algorithm”.

The performance evaluation of the “Classifier Selection Genetic Algorithm” coupled with the mEVE is presented in Figure 2. The “Classifier Selection Genetic Algorithm” starts from the ensemble model with 88.07% training and 76.97% testing accuracy and 67 ELM classifiers. Then, the “Classifier Selection Genetic Algorithm” iteratively increases the quality of the initial set of ELM classifiers and helps build a better ensemble model for each generation. The “Classifier Selection Genetic Algorithm” found the best ensemble model in generation 606 and stopped in generation 673.

The classification performance of the proposed fsEVE approach is compared with similar studies in the literature and is presented in Table 3. An fMRI study by Guo et al. used support vector machine (SVM) for classifying MDD and HCs and obtained an accuracy of 76.42%. Further, this study observed brain changes in the orbitofrontal cortex region [23]. Another study conducted by Ni et al. classified MDD from HCs and obtained an accuracy of 90% using SVM model [24]. Li et al. conducted an fMRI study, used a deep neural network (DNN) model for extracting the features and kernel extreme learning machine (KELM) for classification, and obtained an accuracy of 89% [25]. Yamashita et al. using fMRI images, classified MDD patients with HCs, and obtained an accuracy of 70% by employing the logistic regression (LR) model [10].

The proposed model is also compared with deep learning models. Noman et al. conducted a study on classification of MDD form healthy controls from a total of 477 subjects (250 MDD and 227 HC) using graph autoencoder fully connected neural network (GAE-FCNN) and obtained an accuracy of 65% accuracy [26]. Dai et al. utilized a residual denoising autoencoder (Res-DAE) architecture on a dataset containing 832 MDD patients and 779 healthy controls, resulting in a classification accuracy of 70.0%. When focusing on a subset of 189 recurrent MDD patients and 427 healthy controls, the Res-DAE model attained an accuracy of 75.1%. Further, model highlighted abnormalities in the cerebral cortex and frontal gyri among MDD patients, underscoring the relevance of these brain regions in MDD [27]. Ho et al. conducted a study on classification of MDD with fNIRS dataset of 263 HCs and 251 patients with MDD, including mild to moderate MDD (139) and severe MDD (77) using an interpretable deep learning (DL) model and obtained an accuracy of 80.9%. Additionally, an accuracy of 78.6% is obtained for MDD severity staging [28]. From the literature, it is observed that most of the studies have used smaller datasets and have employed traditional machine learning and deep learning approaches. In convolution neural network (CNN), magnetic resonance images are used directly without extracting features and reasonable accuracy can be achieved by hyperparameter tuning. However, using CNN models, it may be difficult to highlight or rank important brain regions responsible for major depressive disorder.

### 3.2. Identifying Brain Regions Responsible for Major Depressive Disorder

**Figure 3:**
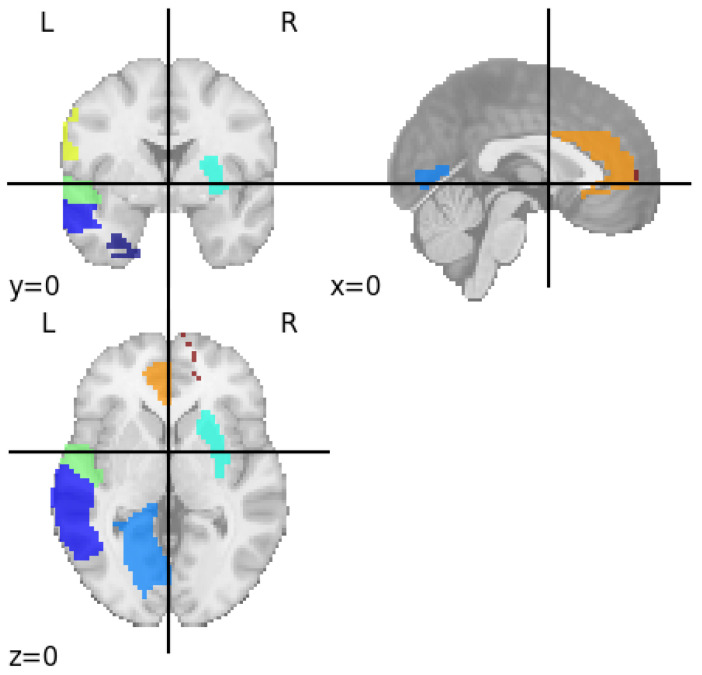
Identified brain regions responsible for major depressive disorder.

The discriminative features obtained from the classifiers with an accuracy of 91.93% are visualized using the automated anatomical labeling template. Nine brain regions are identified namely left superior temporal gyrus, left postcentral gyrus, left anterior cingulate gyrus, right inferior parietal lobule, right superior medial frontal gyrus, left lingual gyrus, right putamen, left fusiform gyrus, and left middle temporal gyrus. Identified brain regions responsible for major depressive disorder is shown in Figure 3 and their functionalities are listed in Table 4.

The superior temporal gyrus (STG) identified in this study is responsible for auditory processing, social cognition, and language comprehension [29]. In several studies, it is reported that MDD patients had smaller cortical volume and surface area in STG than HCs and reduced functional connectivity between the STG and other brain regions is also observed [30,31]. The postcentral gyrus identified from this study forms the primary somatosensory cortex, which is responsible for receiving and integrating sensory information such as touch, pain, temperature, and vibration [32]. In several studies, decreased ReHo in the postcentral gyrus is observed in patients with MDD, reflecting changes in brain regions [33,34]. Additionally, many studies reported that MDD is associated with increased and decreased gray matter volume in the postcentral gyrus, and also the postcentral gyrus often shows hyperactivity during emotion regulation tasks, suggesting altered integration of emotional and sensory information in depression [35,36]. Anterior cingulate cortex (ACC) region identified in this study is involved in a range of cognitive and emotional processes, including attention allocation, emotion regulation, decision-making, error detection, and motivation [37,38]. According to a study conducted by Ibrahim, people with MDD frequently have lower ACC volumes, particularly in the right hemisphere, which is associated with more severe MDD symptoms [39]. The inferior parietal lobule identified in this study is involved in integrating auditory, visual, and somatosensory information and plays key roles in language comprehension, spatial awareness, and body image [40]. Shao et al. reported that MDD patients have reduced resting-state connection between the ACC and inferior parietal lobule, underscoring abnormalities associated with emotional and cognitive processing [41]. The superior medial frontal gyrus, one among the identified brain region in this study, is a key node of the default mode network, primarily involved in self-referential activity [42]. A study by baker et al. reported that the superior medial frontal gyrus has been associated with difficulty disengaging from distressing self-referential thoughts due to abnormal changes in brain networks [43].

Another region identified from this study is the lingual gyrus, which is primarily involved in visual processing, particularly in recognizing letters, encoding visual memories, and analyzing complex images and scenes [44]. Functional studies show that the lingual gyrus is activated during exposure to negative visual stimuli, visual memory tasks, and emotional image processing [45,46]. The putamen identified in this study is primarily responsible for regulating and facilitating movement, contributing to learning, reward, cognitive functions, and addiction [47,48]. Several studies have revealed that, when compared to HCs, people with depression had smaller putamen sizes and anatomical abnormalities [49,50]. Furthermore, functional imaging studies have found changes in putamen, which may be related to impaired reward processing, motivation, poorer external emotion regulation in depression, suggesting difficulties in managing emotional response [49,51]. The fusiform gyrus is mainly in charge of high-level visual processing, involving memory, multisensory integration, color processing, and object, word, face and body recognition [52]. A latest meta-analysis found that patients with MDD exhibited decreased gray matter volume in the left fusiform gyrus compared to those without depression [53]. Another study by Yao et al. reported, in MDD, decreased cortical folding and hypoconnectivity of the fusiform gyrus with sensorimotor areas, along with increased individual variability in functional connectivity between the fusiform gyrus, which correlated positively with depression severity scores [54]. The middle temporal gyrus region, which is identified from this study, is located on the lateral surface of the temporal lobe between the superior and inferior temporal gyri, and it is primarily involved in language and semantic memory processing, visual perception, and multimodal sensory integration [55]. Recent studies have identified significant functional abnormalities of the middle temporal gyrus in MDD. A multi-center study using structural covariance network analysis found that the middle temporal gyrus exhibited volumetric differences between MDD patients and HCs, indicating its involvement in the disorder’s brain architecture [56].

The brain regions identified from this study such as the left superior temporal gyrus, left postcentral gyrus, left anterior cingulate gyrus, right inferior parietal lobule, right superior medial frontal gyrus, left lingual gyrus, right putamen, left fusiform gyrus, and left middle temporal gyrus are the regions that clinical research has repeatedly linked to major depressive disorder. Clinical neuroimaging studies have shown that people with MDD often experience structural and functional changes in these regions, which are involved in mood regulation, emotional processing, and cognitive function [57]. The regions identified from this study, the anterior cingulate and medial frontal gyri, are central to mood and cognitive control, while the putamen and temporal regions are connected to motivation and emotional processing [58,59]. Recognizing these specific brain patterns can help doctors make more accurate diagnoses, as these changes are commonly observed in people with MDD. They can also provide valuable clues about how severe the illness might become or how someone might respond to different treatments, since changes in these regions are linked to treatment outcomes and symptom improvement.

## 4. Conclusions

In this study, feature/sample selective evolving voting ensemble approach is proposed to classify major depressive disorder and healthy controls from functional magnetic resonance imaging. The feature/sample selective evolving voting ensemble is trained using the regional homogeneity features and a testing accuracy of 91.93% is achieved. Using the proposed approach, nine brain regions, namely the left superior temporal gyrus, left postcentral gyrus, left anterior cingulate gyrus, right inferior parietal lobule, right superior medial frontal gyrus, left lingual gyrus, right putamen, left fusiform gyrus, and left middle temporal gyrus, which are responsible for MDD, are identified. The study reveals that these regions are associated with key functionalities of major depressive disorder, such as emotional regulation, self-awareness, and impulsivity. From these findings, it is evident that functional magnetic resonance imaging and ensemble approaches can be used for accurate detection of major depressive disorder.

## Figures and Tables

**Table 1:** Demographic information of the dataset used in the study.

	Healthy Controls	MDD
Number of subjects	1104	1276
Female	642	813
Male	462	463
Average age	36 years Range (12–82)	36 years Range (14–80)

**Table 2:** Performance evaluation of the feature/sample selective evolving voting ensembler (fsEVE).

SI No.	Number of ELM Classifiers	Number of Generations	Max Train/ Test Accuracy (Set of ELM)	Min Train/ Test Accuracy (Set of ELM)	mEVE Train/ Test Accuracy	Train/Test F1 Score
1.	401	193	62.41/54.45	50.6/49.58	98.21/91.09	0.98/0.95
2.	361	799	61.70/57.48	51.78/49.75	97.93/90.76	0.98/0.95
3.	363	943	65.04/51.76	51.27/50.25	97.70/90.92	0.98/0.96
4.	431	408	62.95/55.80	51.28/51.09	99.10/90.59	0.99/0.97
5.	25	673	60.34/53.61	50.93/50.25	99.61/91.93	0.99/0.98

**Table 3:** Performance comparison.

SI No.	Authors	Sample Size	Approach	Accuracy
1.	Guo et al. [23]	MDD-101, HC-49	SVM	76.42%
2.	Ni et al. [24]	MDD-60, HC-60	SVM	90%
3.	Li et al. [25]	MDD-1300, HC-1128	KELM	86%
4.	Noman et al. [26]	MDD-250, HC-227	GAE-FCNN	65%
5.	Dai et al. [27]	MDD-832, HC-779	Res-DAE	70%
6.	Proposed Approach	MDD-1276, HC-1104	fsEVE	91.93%

**Table 4:** Identified brain regions and their functions.

SI No	Regions	Functions
1.	Left Superior Temporal Gyrus	Social cognition, auditory processing and Language comprehension
2.	Left Postcentral Gyrus	Processing sensory information from the skin, muscles, and joints
3.	Left Anterior Cingulate Gyrus	Motivation and goal directed behaviour, cognition, Visuomotor and auditory
4.	Right Inferior Parietal Lobule	Sensory integration, Language, Socialcognition, Visuomotor and auditory processing
5.	Right Superior Medial Frontal Gyrus	Working memory, impulse control, mood regulation and self-awareness
6.	Left Lingual Gyrus	Linguistic processing
7.	Right Putamen	Addiction, Congnitive function and Learning
8.	Left Fusiform Gyrus	Reading and Emotional perception
9.	Left Middle Temporal Gyrus	Language, Visual perception and Semantic memory

## Data Availability

The original data presented in the study are openly available at https://rfmri.org/REST-meta-MDD (accessed on 21 January 2020).

## Abbreviations

The following abbreviations are used in this manuscript:

MDD	Major Depressive Disorder
fMRI	Functional Magnetic Resonance Imaging
ReHo	Regional Homogeneity
HCs	Healthy Controls
fsEVE	Feature/Sample Selective Evolving Voting Ensemble
mEVE	Modified Evolving Voting Ensemble
ELM	Extreme Learning Machine
AAL	Automated Anatomical Labeling
